# Mechanism of the JAK2/STAT3-CAV-1-NR2B signaling pathway in painful diabetic neuropathy

**DOI:** 10.1007/s12020-019-01880-6

**Published:** 2019-03-04

**Authors:** Chuan-Da Li, Jia-Yi Zhao, Jia-Li Chen, Jia-Hui Lu, Mao-Biao Zhang, Qi Huang, Yan-Nan Cao, Gai-Li Jia, Yuan-Xiang Tao, Jun Li, Hong Cao

**Affiliations:** 1 Department of Anesthesiology, Second Affiliated Hospital of Wenzhou Medical University, Pain Medicine Institute of Wenzhou Medical University, 325035 Zhejiang, China; 2 Department of Anesthesiology, New Jersey Medical School, Rutgers, The State University of New Jersey, Newark, NJ 07103 USA

**Keywords:** Painful diabetic neuropathy, JAK2/STAT3, CAV-1, NR2B

## Abstract

**Purpose:**

The aim of the present study was to further elucidate the role of JAK2/STAT3-CAV-1-NR2B on painful diabetic neuropathy.

**Methods:**

In vivo, the mechanical withdrawal threshold and thermal withdrawal latency were measured to evaluate neuropathic pain behaviors (*n=* 8), while western blot (*n=* 5) and an immunofluorescence double staining experiment (*n=* 6) were performed to understand the molecular mechanism. In vitro, the individual culture of BV2 mouse microglia cell lines, the co-culture of BV2 mouse microglia cell lines and PC12 rat neuron cell lines, and western blot analysis were performed to understand the molecular mechanism between microglia and neurons.

**Results:**

The expression of p-JAK2, p-STAT3, t-CAV-1, and p-NR2B was upregulated in the dorsal horn of DNP rats throughout the experiment. Through the immunofluorescence double staining experiment, it was found that p-STAT3 was mainly expressed in activated microglia, and this condition can be stably maintained for approximately 2 weeks after the establishment of the DNP model. The intrathecal injection of JAK2 inhibitor AG490 can relieve the abnormal expression of p-JAK2, p-STAT3, t-CAV-1, and p-NR2B, and relieve pain. The remission of AG490 began on the third day, and it could be stably sustained for 14 days. In vitro high-glucose induced the activation of p-STAT3 in microglia, thereby upregulating the expression of p-CAV-1 and p-NR2B in neurons in the co-culture system. JAK2 inhibitor AG490 can alleviate the abnormal expression of these proteins in the JAK2/STAT3-CAV-1-NR2B signaling pathway in vitro.

**Conclusions:**

Microglial JAK2/STAT3 signaling probably contributes to neuropathic pain by activating the CAV-1-NR2B pathway.

## Introduction

Type-2 diabetic mellitus (T2DM) has reached a pandemic status, and has shown no signs of abatement. Painful diabetic neuropathy (DNP) has been generally considered to be one of the most common complications of T2DM with a 30–50% incidence, and it has also been recognized as one of the most difficult types of pain to treat [1]. Glycemic control and the use of analgesic drugs are the primary treatments for DNP. However, these are accompanied by unobvious curative effects. Exploring the pathogenesis of DNP would be helpful for developing novel therapeutic strategies.

Spinal cord dorsal horn glial cell signal transduction-induced central sensitization is one of the important pathogeneses of DNPs. The activation of microglia produces and releases a variety of cytokines and excitatory substances of neurons or glial cells [2–4], promotes NR2B activation on neuronal cells, NR2B is a subunit on NMDA, and increases NMDA-mediated current. NMDA receptor activation can further induce the sensitization of the central nervous system, which is one of the important pathogeneses of DNP [5, 6].

JAK2/STAT3 signal transduction, as a classical intracellular signal transduction pathway, participates in many pathophysiological processes, such as cell differentiation, proliferation, inflammation, and pain formation. The activation of microglia caused by JAK2/STAT3 has a significant effect on neuropathic pain. After nerve injury induces vigorous IL-6 production in dorsal root ganglion (DRG), the cytokines may be transported to central terminals of primary afferents. The released cytokines stimulate the JAK/STAT3 signaling pathway in spinal microglia and promote genesis of neuropathic pain. Meanwhile, these cytokines further activate glial cells and neurons to release more activating substances such as ATP, pro-inflammatory factors, reactive oxygen species (ROS), nitric oxide (NO), prostaglandins (PGs), etc. These activating substances further enhance neuropathic pain [7]. In addition, JAK2/STAT3 signal transduction has an effect on spinal NMDA-induced currents, causing neuropathic pain [8]. However, the relationship between the JAK2/STAT3 pathway and activation of microglia in DNP remains unclear.

Caveolae are a specialized type of lipid raft that are stabilized by oligomers of the caveolin protein. Caveolin-1 (CAV-1), a major protein component of caveolae, is an important gene targeting for STAT3. The overexpression of p-STAT3 can cause CAV-1 promoter activation and increase gene expression [9]. CAV-1 regulates neuronal plasticity and receptor transport to regulate NR2B-NMDAR, which is closely correlated with pathologic pain and central sensitization [10].

However, no studies on DNP have focused on the JAK2/STAT3 signaling pathway. Therefore, the present study further explored the correlation between DNP and microglial activation by combining in vivo and in vitro experiments to explore the role of this signaling pathway in the pathogenesis of DNP, and explore its regulation of the downstream of CAV-1 and NR2B.

## Materials and methods

### Animals

**Fig. 1:**
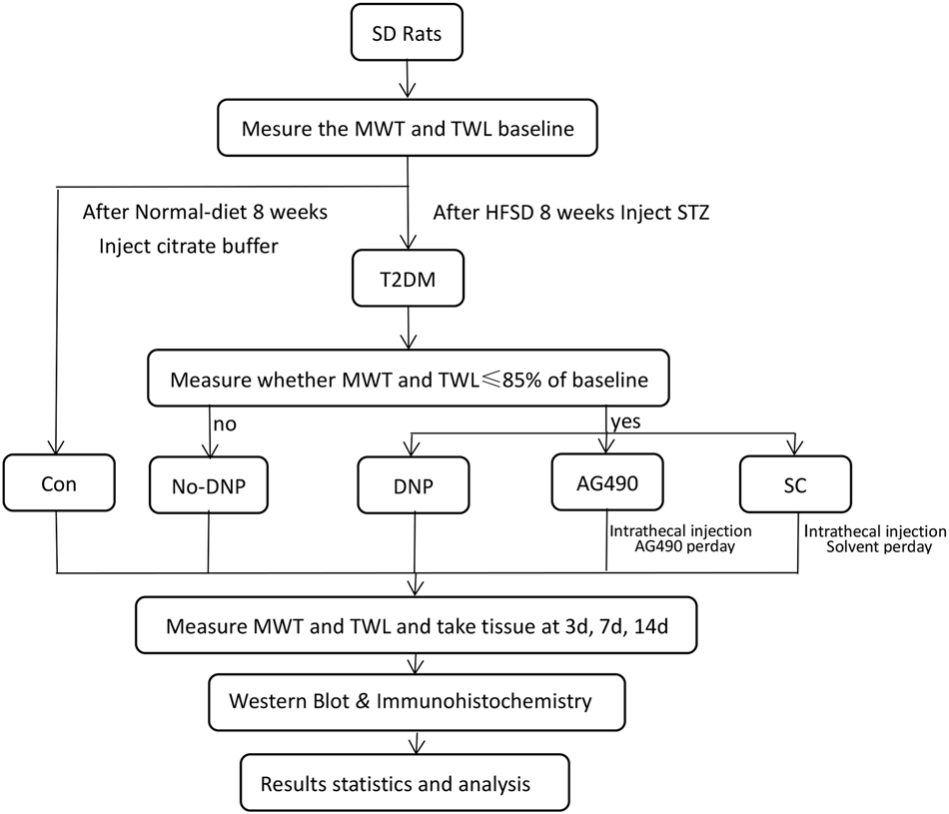
In vivo experiment design scheme. After 3 days of injection, conditions with blood glucose levels ≧ 16.7 mmol/L were considered as T2DM. Rats in the AG490 group and dimethylsulfoxide (DMSO) group were injected, respectively, with 10 μL (1 mmol/L) of AG490 and 10 μL of 3.5% DMSO once a day for 14 days. Mechanical withdrawal threshold (MWT) and thermal withdrawal latency (TWL) were measured before streptozocin (STZ) injection, on day 3 after STZ injection (as a reference for successful modeling) and on days 3, 7, and 14 after intrathecal injection (*n=* 5)

**Fig. 2:**
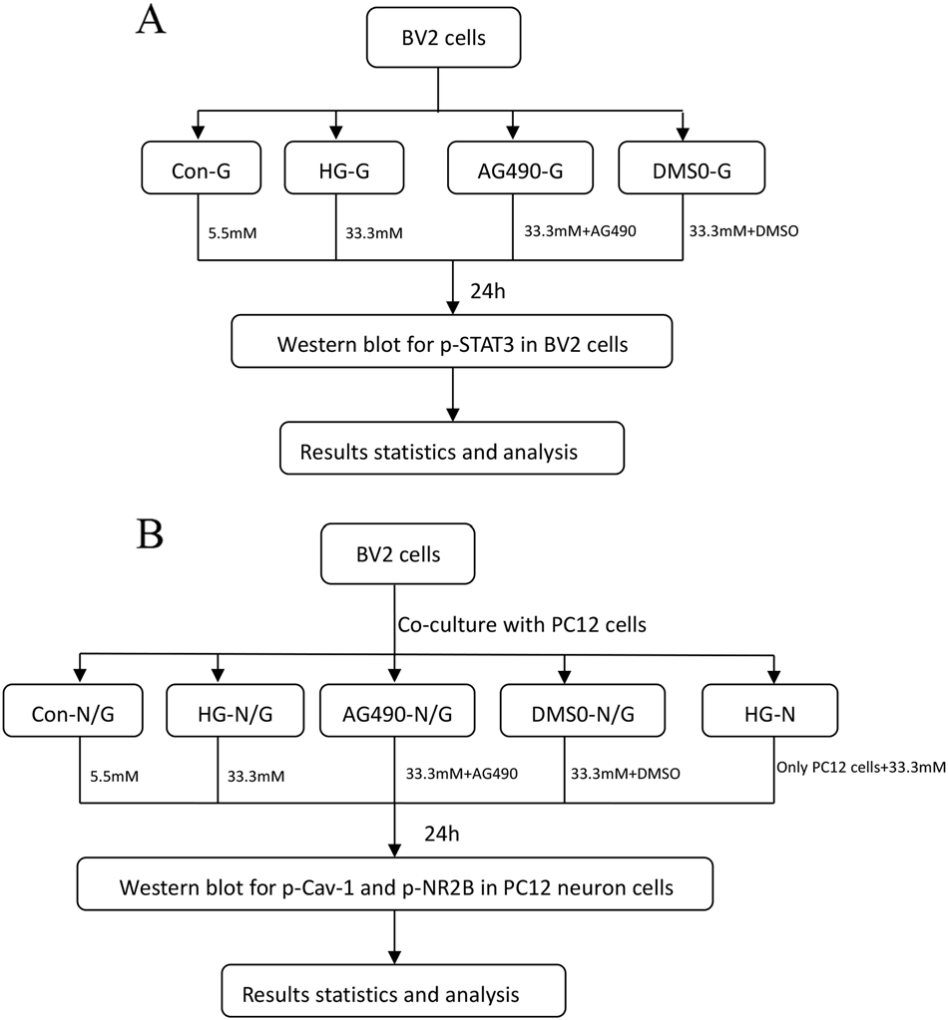
In vitro experiment design scheme. **A** Step 1 BV2 cells were seeded at 1 × 10^6^ cells/well in a 6-well plate. Con-G: BV2 cells treated with low glucose (5.5 mM d-glucose); HG-G: BV2 cells treated with high glucose (33.3 mM d-glucose); AG490-G: BV2 cells treated with AG490 (50 μM) + high glucose; DMSO-G: BV2 cells treated with dimethylsulfoxide (DMSO) + high glucose. After 24 h of culture, western blot was used to detect the expression of p-STAT3 in BV2 microglia cells. **B** Step 2 Transwell was used to establish a co-culture system of BV2 cells and PC12 cells. Con-N/G: BV2 cells and PC12 cells treated with low glucose (5.5 mM d-glucose); HG- N/G: BV2 cells PC12 cells treated with high glucose (33.3 mM d-glucose); AG490- N/G: BV2 cells PC12 cells treated with AG490 (50 μM) + high glucose; DMSO-N/G: BV2 cells PC12 cells treated with DMSO + high glucose. HG-N: PC12 cells treated with low glucose. After 24 h of culture, western blot was used to detect the expression of p-Cav-1 and p-NR2B in PC12 cells

The study protocol was approved by the Animal Research Committee of Wenzhou Medical University. All animal experiments were performed in accordance with the National Institutes of Health Guidelines for the Care and Use of Laboratory Animals, and in accordance with the Animal Research: Reporting In Vivo Experiments (ARRIVE) guidelines. Sprague-Dawley (SD) rats, weighing 120–160 g, were provided by the Center for Laboratory Animals of Wenzhou Medical University (License No. WYDW2014-0015). These rats were housed in room temperature, which was maintained within 23–25 °C, allowed free access to food and water, and placed under a 12-hour/12-hour dark/light cycle. The detailed design schemes were depicted in Figs 1 and 2.

### Induction of T2DM and DNP

These T2DM models were established, as previously described [11, 12]. After 2 weeks of adaptive feeding, these rats were randomized into two groups: Con group and T2DM group. Rats in the T2DM group were fed with a high-fat-sugar diet (HFSD) for 8 weeks, while rats in the Con group were fed with a normal-diet. After 8 weeks, the insulin sensitivity trail was performed by testing for fasting blood glucose and insulin on blood samples collected from the tail vein. Insulin sensitivity index (ISI) = 1/(blood glucose × insulin). These results revealed that there was a significant difference in ISI between these two groups (*P<* 0.05), implying that insulin resistance was successfully induced in the T2DM group. Subsequently, rats in the T2DM group were intraperitoneally injected with streptozocin (STZ) (Sigma Co., St. Louis, MO, USA) at a dose of 35 mg/kg. As a control, rats in the Con group were intraperitoneally injected with the same dose of citrate buffer. After three days of injection, rats in the T2DM group had a caudal vein fasting blood glucose of ≥16.7 mmol/L, and were considered as T2DM rats. After measuring the mechanical withdrawal threshold (MWT) and thermal withdrawal latency (TWL) of these rats, rats with MWT and TWL values ≤ 85% of baseline (before the high-fat-sugar diet) were considered as a successful model of type-2 painful diabetic neuropathy, and was redefined as the T-DNP group. The remaining rats in the T2DM group were redefined as the No-DNP group.

### Subarachnoid catheterization and AG490 administration

AG490, a specific inhibitor of JAK2, inhibits the activation of STAT3 by selectively blocking JAK2. AG490 (1 mmol/L) was dissolved in 3.5% dimethylsulfoxide (DMSO), and 3.5% DMSO was used as the control vehicle (DMSO group). Rats in the T-DNP group were randomly divided into three groups: DNP group, AG490 group, and DMSO group. Rats in the AG490 and DMSO groups received subarachnoid catheterization, as previously described. Under anesthesia with chloral hydrate (0.3 g/kg), a PE-10 silastic tubing (Ningbo Science and Technology Park to the Software Technology Co., Ltd, China) was intrathecally inserted between the L4 and L5 vertebrae, and advanced 2 cm into the lumbar enlargement of the spinal cord. The external end of the intrathecal catheter was tunneled under the skin to the neck area, and the outer part of the catheter was exposed, carefully plugged and fixed onto the skin. The animals were allowed to recover for 5–6 days after the surgery. In order to verify the location of the catheter, 10 μL of 2% lidocaine was given through the catheter. Limb paralysis response without neurological deficits indicated that the insertion was successful. Then, the rats in the AG490 group and DMSO group were injected, respectively, with AG490 10 μL (1 mmol/L) and 3.5% DMSO 10 μL once a day for 14 days.

### Behavioral tests

MWT and TWL were measured before STZ injection, on day 3 after STZ injection (as a reference for successful modeling) and on days 3, 7, and 14 after intrathecal injection. The detained behavioral tests and model preparation were described in the Supplementary Methods.

### Cell cultures and treatments

BV2 immortalized murine microglia cell line was purchased from MX Biotechnology Company (Shanghai, China). PC12 neuronal cell line cells were provided by the College of Pharmacy of Wenzhou Medical University. The cells were cultured in Dulbecco’s modified eagle’s medium (DMEM) supplemented with 10% fetal bovine serum (FBS), 100 U/mL of penicillin, 100 μg/mL of streptomycin, and 5.5 mmol/L of glucose.

### BV2 microglia cell line cultures and treatments

BV2 cells were seeded at 1 × 10^6 ^cells/well in a 6-well plate. BV2 microglia cells were randomly divided into four groups: control group (Con-G group), high-glucose group (HG-G group), JAK2 inhibitor group (AG490-G group), and solvent control group (DMSO-G group). BV2 cells were cultured with low-glucose medium (5.5 mM d-glucose) to the exponential phase. Then, BV2 cells were replaced with low-glucose medium (5.5 mM d-glucose), high-glucose medium (33.3 mM d-glucose), AG490 (50 μM) plus high-glucose medium (33.3 mM d-glucose), and DMS plus high-glucose medium (33.3 mM D-glucose), respectively. After 24 h of culture, western blot was performed to detect the expression of p-STAT3 in BV2 microglia cells.

### BV2 microglia cell co-culture with PC12 neuron cells

Transwell was used to build the co-culture system of BV2 microglia cells and PC12 neuron cells. These cells were randomly divided into five groups: control group (Con-N/G group), high-glucose group (HG-N/G group), JAK2 inhibitor group (AG490-N/G group), solvent control group (DMSO-N/G group), and neuron high-glucose group (HG-N group). In the HG-N group, PC12 cells were seeded in a six-well plate with no cells seeded in the transwell, which was inside the six-well plate. For the other four groups, PC12 cells were seeded in a six-well plate, and BV2 cells were seeded in the transwell, which was inside the six-well plate. Cells in the Con-N/G group, HG-N/G group, AG490-N/G group, DMSO-N/G group, and AG490-N/G group were cultured with low-glucose medium (5.5 mM d-glucose) to the exponential phase, and this was subsequently replaced with low-glucose medium (5.5 mM d-glucose), high-glucose medium (33.3 mM d-glucose), AG490 (50 μM) plus high-glucose medium (33.3 mM d-glucose), DMSO plus high-glucose medium (33.3 mM d-glucose), and high glucose (33.3 mM d-glucose), respectively. After 24 h, western blot analysis was carried out to detect the expression of p-CAV-1 and p-NR2B in PC12 cells.

### Western blot analysis

Protein samples were separated using 10% sodium dodecyl sulfate polyacrylamide gel electrophoresis (SDS-PAGE, 60 g of total protein per lane) and transferred onto a polyvinylidene fluoride membrane (Merck Millipore, Temecula, CA, USA). In addition, gels stained with Coomassie Blue were used to confirm the equal amounts of protein loaded on each lane. The membranes were incubated overnight at 4 °C with primary polyclonal rabbit anti-p-CAV-1 (1:500; Santa Cruz Biotechnology, Santa Cruz, CA, USA), anti-t-CAV-1 antibody (1:500; Santa Cruz Biotechnology, Santa Cruz, CA, USA), anti-p-JAK2 (1:500; Abcam, Cambridge, MA, USA), anti-p-STAT3 (1:1000; Cell Signaling Technology, Danvers, MA, USA), or anti-p-NR2B antibody (1:500; Millipore, Billerica, MA, USA). The membranes were extensively applied with Tris buffered saline Tween 20 (TBST) and incubated for 2 h with horseradish peroxidase conjugated secondary antibody (1:3000; Abcam, UK)) at room temperature. The immune complexes were detected using a nitro blue tetrazolium/5-bromo-4-chloro-3-indolyl phosphate assay kit (Sigma Co., St. Louis, MO, USA), or chemiluminescence (Pierce, Waltham, MA, USA). The density of the specific bands was analyzed using NIH ImageJ software, and the expression levels of these proteins were normalized to β-actin.

### Immunohistochemistry

Rats were deeply anaesthetized with 400 mg/kg of 5% chloral hydrate, transcardially perfused with 200 mL of phosphate buffered saline (PBS, 0.01 M; Solarbio Science & Technology, Beijing, China), followed by 300 mL of freshly prepared 4% paraformaldehyde in 0.01 M PBS. The fourth to sixth lumbar segments of the spinal cord were removed, post-fixed with the same fixative for 12 h at 4 °C, and placed in 10%, 20 and 30% (w/v) sucrose solutions, one by one, for 12 h at 4 °C. Then, the dehydration spinal cord tissue was embedded with OTC, 20 μm was cut by a frozen section machine at a constant temperature of −20 °C, washed with 0.01 M PBS solution at 4 °C three times for 20 min each time, and incubated in a blocking solution (0.2% Trition; 3% goat serum; 0.01 M PBS) for 1 h at 26 °C. Then, the tissue was incubated for 24 h at 4 °C with the following primary antibodies: p-STAT3 (1:200; Pierce, Waltham, MA, USA), GFAP (1:1000; Merck Millipore, Temecula, CA, USA), OX-42 (1:200; Abdserotec, Hercules, CA, USA), and Neu (1:800; Merck Millipore, Temecula, CA, USA). After incubation, the sections were placed in room temperature for 1 h, washed three times (10 min each time) with 0.01 M PBS solution at 4 °C, and incubated for 2 h at 26 °C with the following secondary antibodies: Alexa Fluor® 546 Goat Anti-Mouse IgG, and Alexa Fluor® 488 Goat Anti-Rabbit IgG (1:1,000; Invitrogen, Waltham, MA USA). Then, the tissues were washed with 0.01 M PBS solution at 4 °C three times at 20 min each time. The slices were observed by fluorescence microscopy and analyzed by Image-Pro Plus software.

### Data analysis

Data were presented as mean ± standard deviation (SD). The results were statistically analyzed using one-way analysis of variance (ANOVA), or paired or unpaired Student’s *t*-test. When the ANOVA results revealed a significant difference, pairwise comparisons between means were tested by the least significant difference method (LSD). These data were analyzed by SPSS 19.0 (SPSS Inc., Chicago, IL, USA). *P* < 0.05 was considered statistically significant.

## Results

### Changes in blood glucose level, insulin level, and insulin sensitivity index

**Fig. 3:**
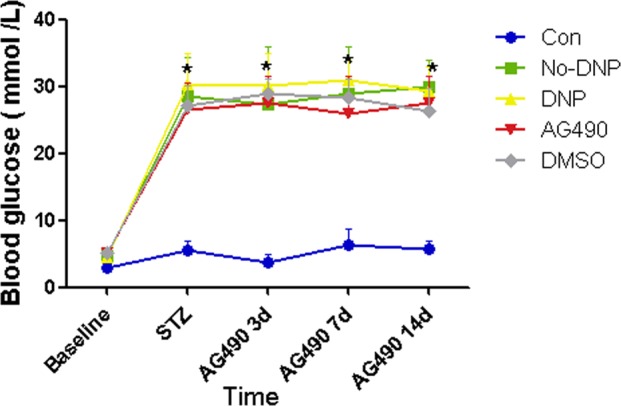
Comparison of blood glucose between groups (Mean ± SD, *n=* 8). The comparison of blood glucose before and at 3 days after administration of streptozocin (STZ) and subarachnoid administration at 3,7,14 days (*n=* 8), ^*^P< 0. 05 vs. Con group

After 8 weeks of HFSD, insulin resistance was successfully elicited. Compared with the Con group, rats in the T2DM group had a higher blood glucose level (*P* < 0.05). However, this was not high enough to meet the diagnostic criteria of T2DM ( > 16.7 mmol/L), although rats in the T2DM group had a higher insulin level and lower insulin sensitivity index (*P* < 0.05, Table 1). At three days after STZ injection, the levels of blood glucose in diabetic rats dramatically increased, when compared with control rats, and this high blood glucose level was maintained until the end of the experiments (*P* < 0.05, Fig. 3). The average levels in the diabetic rats were higher than 16.7 mmol/L, indicating that the type-2 diabetic models were successful established.

**Table 1:** Comparison of blood glucose, insulin level, and insulin sensitivity index (ISI) after normal-diet (Con) or a high-fat-sugar diet (T2DM) (Mean ± SD, *n=* 8)

Group	Blood glucose levels (mmol/L)		Insulin levels (mIU/L)		ISI	
	Baseline	After high-fat-sugar diet (HFSD)	Baseline	After HFSD	Baseline	After HFSD
Con	2.89 ± 0.47	4.60 ± 0.54	7.51 ± 2.00	9.36 ± 2.87	−3.22 ± 0.41	−3.15 ± 0.43
T2DM	3.65 ± 1.23	5.10 ± 0.90	8.01 ± 2.44	27.78 ± 6.09^*^	−3.08 ± 0.56	−4.51 ± 0.39^*^

^*^P < 0.05 vs. Con group

### MWT and TWL

**Fig. 4:**
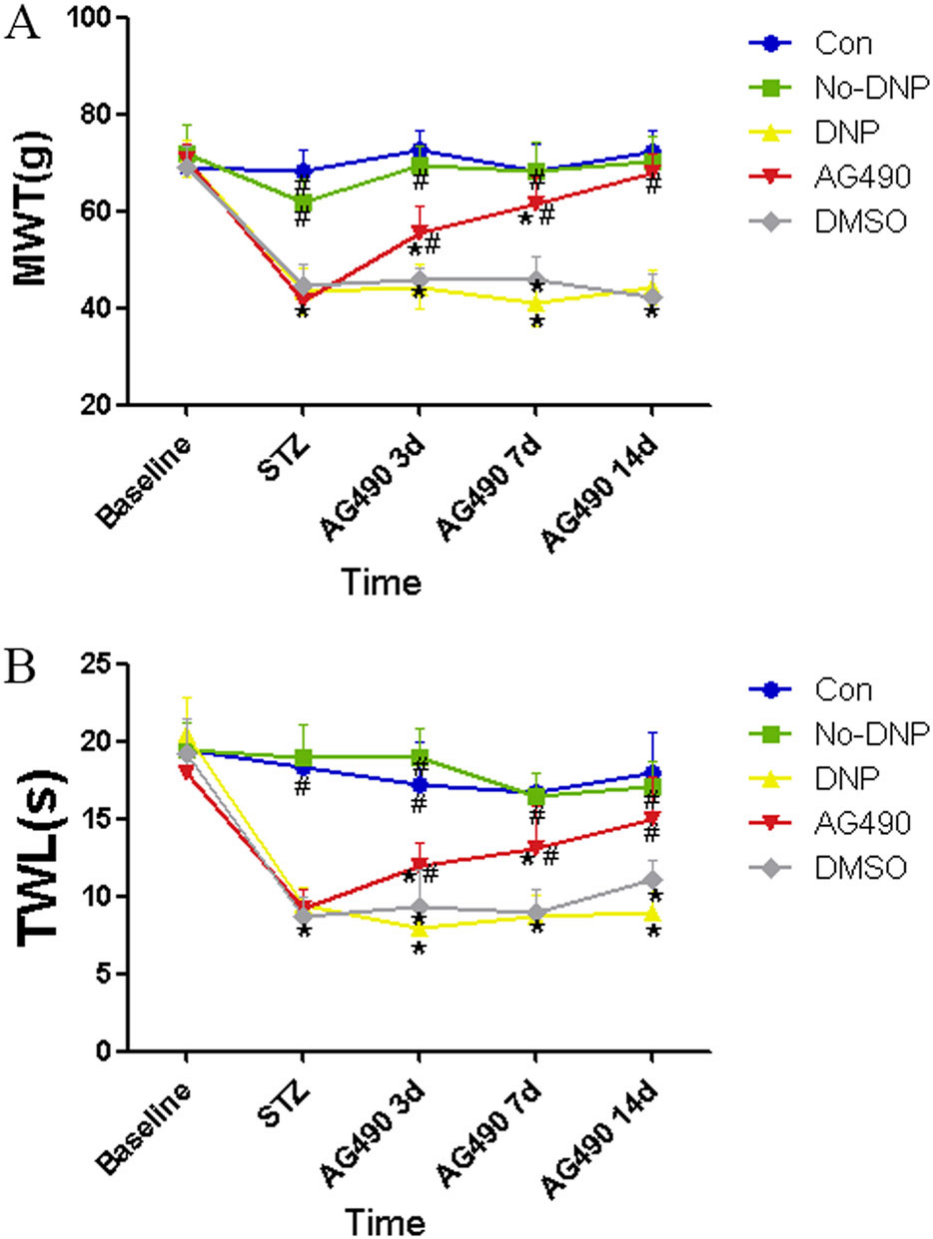
Comparison of mechanical withdrawal threshold (MWT) and thermal withdrawal latency (TWL) between groups. **A** The comparison of MWT before and at 14 days after administration of streptozocin (STZ) and subarachnoid administration at 3, 7, 14 days (*n=* 8). *P < 0.05 vs. Con group, #*P* < 0.05 vs. painful diabetic neuropathy (DNP) group. **B** The comparison of TWL before and at 14 days after administration of STZ and subarachnoid administration at 3, 7, 14 days (*n=* 8). *P < 0.05 vs. Con group, #*P* < 0.05 vs. DNP group

Compared with the Con group, the MWT and TWL of the DNP and DMSO groups both significantly decreased (*P* < 0.05). Compared with DNP group, the MWT and TWL of the AG490 group increased after AG490 intrathecal administration (*P* < 0.05). The reductions in MWT and TWL were time-dependently reversed in type-2 DNP rats after the administration of AG490. These results indicate that the injection of AG490 alleviated the mechanical hyperalgesia and thermal allodynia in type-2 DNP rats. Furthermore, no significance difference was found between the Con and no-DNP groups (Fig. 4).

### The JAK2/STAT3-CAV-1-NR2B signal pathway was activated in the spinal dorsal horn of DNP and could be inhibited by AG490 in vivo

**Fig. 5:**
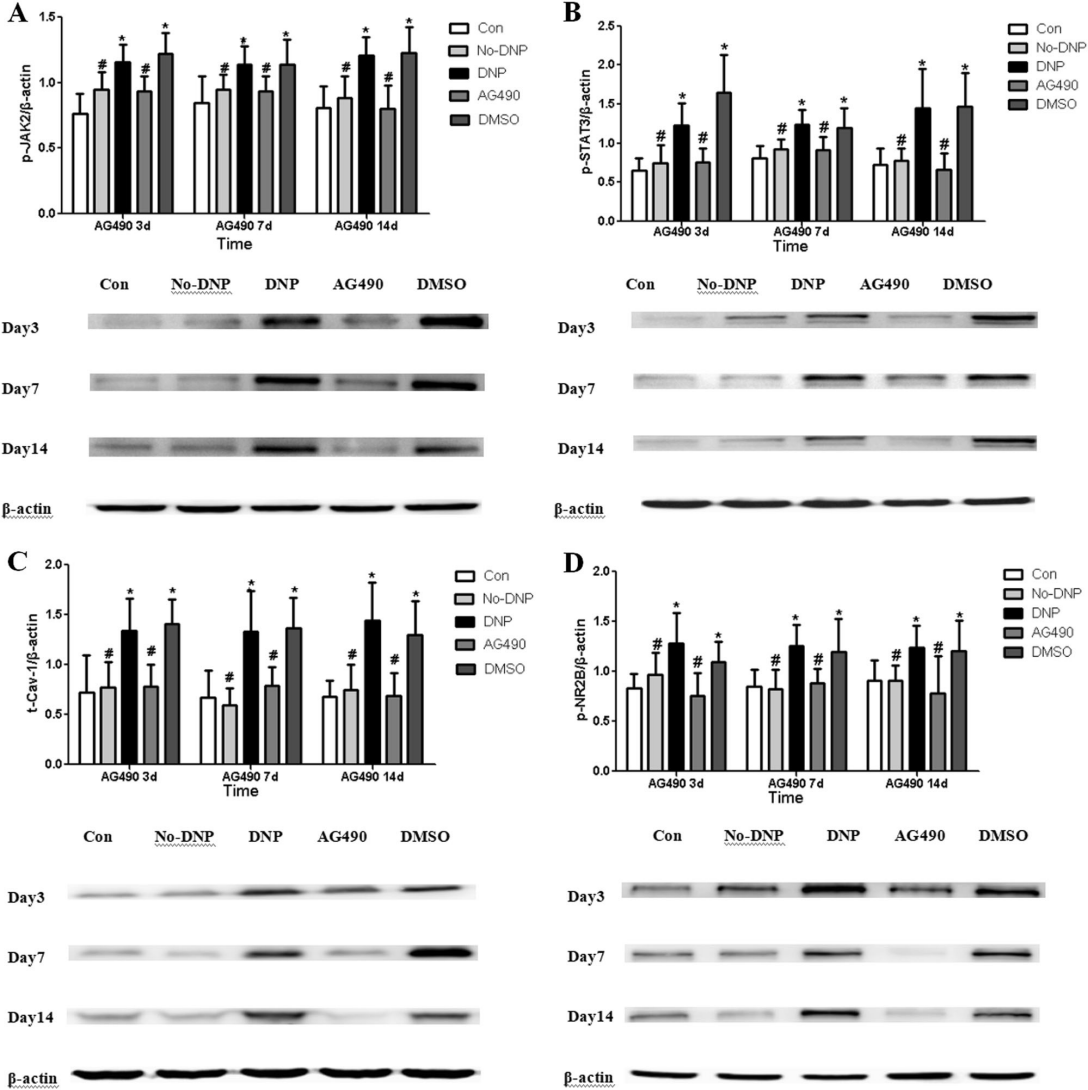
The representative immunoblotting bands (bottom) and the quantitative data (top) showed following points. (Bottom of **A–D**): The expression of p-JAK2, p-STAT3, t-Cav-1 and p-NR2B in spinal cord of group Con, No-DNP, DNP, AG490 and DMSO animals by western blotting experiment. (Top of **A–D**): The western blots analysis for the p-JAK2, p-STAT3, t-CAV-1, and p-NR2B protein (*n=* 5/group) in the different group at different time point. *P < 0.05 vs. Con group; #*P* < 0.05 vs. Painful diabetic neuropathy (DNP) group

Compared with the Con group, the levels of p-JAK2, p-STAT3, t-CAV-1, and p-NR2B in the DNP group significantly increased (*P* < 0.05). As shown in Fig. 5, the administration of AG490 blocked the increase in p-JAK2, p-STAT3, t-CAV-1, and p-NR2B levels in the spinal dorsal horn of type-2 DNP rats (*P* < 0.05). However, there was no significant difference between the DNP and DMSO groups at any time point (*P* > 0.05, Fig. 5).

### The p-STAT3 mainly co-labeled with microglial cells in the spinal cord of DNP in vivo

**Fig. 6:**
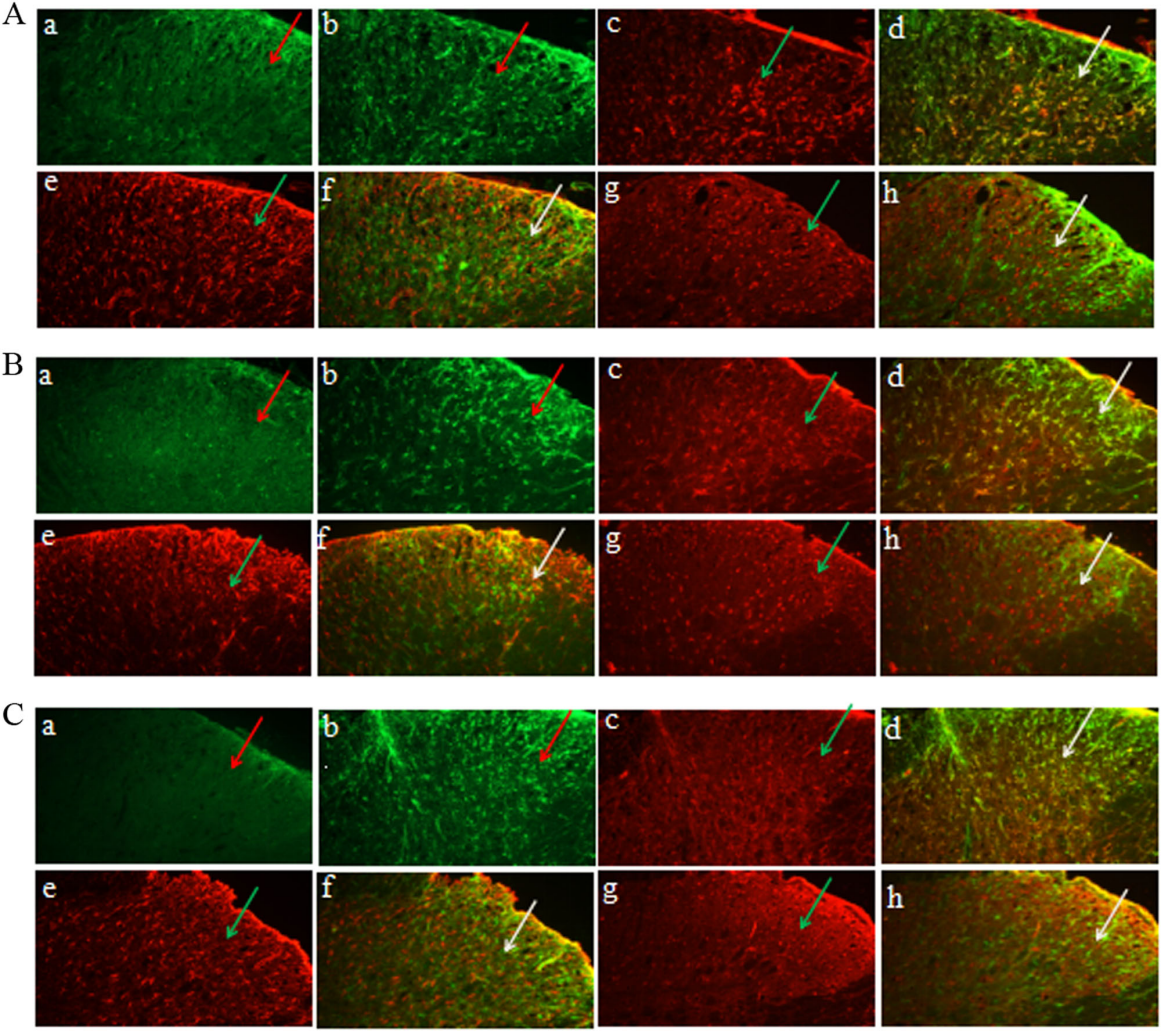
The expression of p-STAT3-IR and localization of p-STAT3 mainly with microglia, but astrocytes and neuron, in the spinal cord dorsal horn of the type-2 painful diabetic neuropathy (DNP) rats. **A** The third day after AG490 intrathecal administration. **B** The seventh day after AG490 intrathecal administration. **C** The fourteenth day after AG490 intrathecal administration. There are numerous p-STAT3-IR exists in the superficial and medial laminae (I-V) of spinal cord horn at group DNP in picture Aa-b, Ba-b, Ca-b (the arrows to the bright green in the pictures). The microglia cells (OX-42, red arrows highlight) and the co-label of p-STAT3-IR with microglial cells (arrow highlights in yellow), astrocytes cell markers GFAP (the arrows to the red highlight of GFAP, yellow highlights for the co-label) and neurons markers NeuN (the arrows to the red highlight of NeuN, yellow highlights for co-label) by double immunofluorescence table experiments, respectively (pictures Ac-f, Bc-f, Cc-f). Neither astrocytes nor neurons in spinal cord of DNP animals are co-labeled with p-STAT3-IR at the third, seventh, and fourteenth days. Three independent experiments were performed with different animals from each experimental group for immunohistochemical experiments (*n=* 6). Tissue slices, 20 μm. Scale bar, 100 μm

In order to examine which types of cells in the dorsal horn of type-2 DNP rats expressed p-STAT3, double-labeled immunofluorescent staining was carried out. The result revealed that p-STAT3 accumulated mainly in the superficial and medial laminae (I-V) of the spinal cord horn, and more p-STAT3-positive cells were found in the DNP group (Fig. 6Aa-b, Ba-b, Ca-b). Immunofluorescence double staining experiments were performed to identify the cell types. Compared with astrocytes and neurons, p-STAT3-IR mainly coexisted with the microglia (Fig. 6Ac-h, Bc-h, Cc-h).

### High-glucose induced the activation of p-STAT3 in BV2 cells and upregulated the expression of p-CAV-1 and p-NR2B in PC12 cells, which could be inhibited by AG490 in vitro

**Fig. 7:**
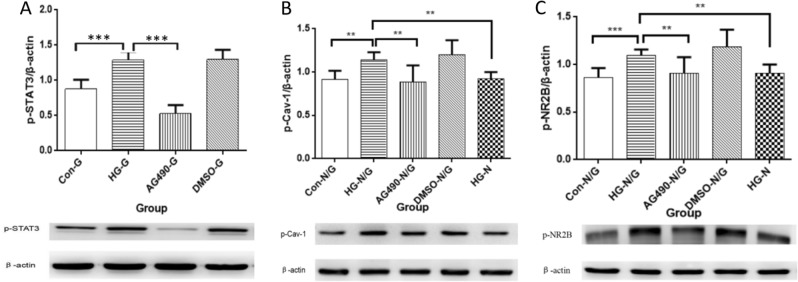
The results of cell culture in vitro. **A** The expression of p-STAT3 in BV2 cells under high glucose. Mean ± SD. *n=* 3. ***P < 0.001. **B** The expression of p-CAV-1 in PC12 cells under co-culture system. Mean ± SD. *n=* 3. **P < 0.01. **C** The expression of p-NR2B in PC12 cells under co-culture system. Mean ± SD. *n=* 3. **P < 0.01, ***P < 0.001

The results of the BV2 cell culture revealed that the expression of p-STAT3 in BV2 cells was upregulated in the HG-G group, when compared with the Con-G group (*P* < 0.001). Moreover, compared with the HG-G group, the expression of p-STAT3 was downregulated in the AG490-G group (*P* < 0.01). However, there was no significant difference between the HG-G and DMSO-G groups. The results of the co-culture system revealed that the expression levels of p-CAV-1 and p-NR2B in PC12 cells were upregulated in the HG-N/G group, when compared with the Con-N/G and HG-N groups (*P* < 0.01). However, when compared with the HG-N/G group, the expression levels of p-CAV-1 and p-NR2B were downregulated in the AG490-N/G group (*P* < 0.01), and there was no significant difference between the HG-N/G and DMSO-N/G groups (Fig. 7).

## Discussion

In the present study, diabetic rats were induced not only by STZ injection, but also by combining with HFSD feeding, in order to elicit insulin resistance, which would better reflect the pathophysiological process of T2DM. Moreover, the subsequent induced DNP rat model could steadily be maintained for approximately 2 weeks both in pain threshold and blood glucose.

### JAK2/STAT3 and the activation of microglia

JAK2/STAT3 signal transduction is involved in the activation of glial cells and formation of neuropathic pain [13]. In the CCI model, the presence of neuropathic pain was accompanied by the high expression of JAK2 and STAT3, and the inhibition of the expression of these factors could significantly relieve pain [14]. Ion channel dysfunction is central to the pathogenesis of painful DNP. In hippocampal neurons, the JAK/STAT signaling pathway is closely correlated to NMDAR function. IL-6 regulates the Ca^2+^ influx of NMDAR through the JAKs/STATs signal pathway, resulting in neurodegenerative lesions [15]. The activation of JAK2 further activates STAT3, and activated stat3 forms a dimer, which is involved in the regulation of the transcription of inflammatory factor genes, possibly acting on neuronal NMDARs, and causing central sensitization, leading to DNP. In the present study, it was found that JAK2 activity increased in DNP rats, while its inhibitor AG490 could downregulate p-JAK2 and p-STAT3 in vivo and in vitro, and relieve pain.

Signal transducers and activators of transcription (STAT) is a unique protein that can induce the expression of target genes [16]. In recent years, studies have found that the activation of STAT3 subtypes is involved in the transmission and modulation of nociceptive information in spinal cord levels after peripheral injury [7]. In the SCI model, and early in the spinal nerve injury, the activation of the JAK2/STAT3 pathway was found in activated microglia [17].

The microglia and astrocytes were both considered to be involved in neuropathic pain, and a researcher supposed that microglia participates in the early phase of pain, while astrocytes contribute to the later phase. The activation of microglia can transmit the abnormal pain signal to neurons of the spinal cord, thereby contributing to the development of neuropathic pain [18]. In spinal nerve ligation-induced pain and bone cancer-induced pain, the activation of microglia is present, and the spinal inhibition of microglia by minocycline effectively reduced allodynia and hyperalgesia [19, 20]. DNP is associated with a slower rate of deafferentation compared with traumatic neuropathy, but, recently, microglia cells also play a role in the development of neuropathic pain in DNP [21]. In the present study, the activation of microglia and astrocytes were both observed in DNP in vivo.

STAT3 mainly expressed in microglia is one of the marker proteins of central nervous system injury. It plays a role in regulating the cytokine-mediated signaling pathway [22]. In the context of maladaptive plasticity, abnormal signaling between glia and neurons also has a role in pain. After traumatic nerve injury, microglia in the dorsal horn of the spinal cord release factors such as brain-derived neurotrophic factor (BDNF), leading to the amplification of nociceptive synaptic processing, resulting in “gating” of neuropathic pain [23]. In the CCI model, and in the early-stage of pain, the double-label immunofluorescence assays revealed that p-STAT3 was mainly co-localized with microglia [7]. However, some studies have indicated that STAT3 co-exists with astrocytes, and participates in the activation of astrocytes [13, 24, 25]. In the present study, the double-label immunofluorescence assays revealed that p-STAT3 was mainly expressed in microglia in the spinal cord, which was not consistent with some of the conclusions above. This may be due to the different model of neuropathic pain, such as the particularity of the hyperglycemia state in DNP model. Moreover, it was found that the high-glucose environment upregulated the expression of p-STAT3 in microglia, which was consistent with what was found in vitro.

### The correlation between CAV-1 and STAT3

Caveolae is a specialized type of lipid raft stabilized by oligomers of caveolin protein. Caveolae has a large number of membrane-bound proteins, which are mostly signal molecules with lipid modification. Caveolae is a platform for the exchange of these signaling molecules, allowing the signal pathways to interact with each other. CAV-1 is the crucial adjustment of the signal pathways in each platform. In general, CAV-1 has a negative regulatory signal transduction pathway for signaling molecules in Caveolae, and it also has the function of enhancing the signal [26–28].

CAV-1 has been shown to directly interact with STAT3 on lipid rafts [29, 30]. Activated STATs leave a lipid raft into the cell, and the subsequent cross-cytoplasm transport requires a specific chaperone protein, in which CAV-1 is one of the chaperone proteins [31]. Research has shown that the JAK/STAT signaling pathway locates on the caveolae containing CAV-1 [32, 33]. CAV-1 is an important gene targeting of STAT3, and STAT3 directly affects the transcription of CAV-1 by directly binding to the CAV-1 promoter. The overexpression of p-STAT3 causes CAV-1 promoter activation and the increase in gene expression [9].

In the present study, in the dorsal horn of the spinal cord, the expression of t-CAV-1 was upregulated in vivo. Owing to laboratory conditions, the expression and distribution of p-CAV-1 in the dorsal horn of the spinal cord were not detected in the present study. In the high-glucose environment, the expression of p-STAT3 was upregulated in BV2, and the expression of p-CAV-1 was upregulated in the co-culture of BV2 and PC12. In contrast, the expression of p-CAV-1 was not upregulated in PC12 alone. Hence, it can be speculated that p-CAV-1 may be a downstream target of p-STAT3. In addition, in vivo and in vitro experiments have confirmed that AG490 can relieve the abnormal expression of CAV-1. In view of the exact relationship with the JAK/STAT signaling pathway, the above-mentioned inference can be further confirmed. However, since the present study did not directly interpret the STAT3-CAV-1 signaling pathway, further experiments were needed to confirm whether CAV-1 acts as an important downstream target of the JAK/STAT pathway in the pathogenesis of DNP.

### The correlation between CAV-1 and NR2B

The central sensitization caused by NMDAR plays an important role in the formation and development of DNP. A number of studies have shown that NR2B activation and neuropathic pain are closely correlated to its production and maintenance [34, 35].

HEAD BP found a close association between CAV-1 and NR2B in the primary culture of rat cortical neurons [31]. CAV-1 regulates neuronal plasticity and receptor transport, regulating NR2B-NMDAR, which is closely correlated to neuropathic pain and central sensitization. In the CCI model, the expression levels of CAV-1 and NR2B in the anterior cingulate cortex were significantly higher than those in normal rats. CAV-1 promoted the expression of NR2B on the membrane, and opened the ion channel, thereby altering neuronal synaptic plasticity, and causing central sensitization. In addition, the increase in NR2B expression can be inhibited by CAV-1 siRNA or NR2B inhibitors. The co-immunoprecipitation and two-hybrid assay also demonstrated a direct interaction between CAV-1 and NR2B. CAV-1 regulated chronic neuropathic pain by modulating NR2B in the anterior cingulate gyrus [10].

In the present study, it was found that the expression of p-NR2B in the dorsal horn of DNP rats was upregulated. In the in vitro experiments, it was also found that the high-glucose environment could increase the expression of p-NR2B in neurons. As the present study did not intervene with the CAV-1-NR2B pathway, it was difficult to elucidate the correlation between these two in the pathogenesis of DNP. In addition, the in vivo and in vitro experiments confirmed that AG490 can alleviate the abnormal expression of p-NR2B, in view of the important position of NMDA in the pathogenesis of neuropathic pain. Furthermore, to a certain extent, it can reflect the significance of the JAK2/STAT3 signaling pathway in DNP treatment.

### Influence of high-glucose on the JAK2/STAT3-CAV-1-NR2B signaling pathway

Persistent high blood glucose level in diabetes plays an initiation role in the change of voltage gated calcium channel and voltage gated sodium channel in the cytomembrane of neuron axons, and the release of neural growth active substances and P substances [36]. This thereby upregulates the excitability of peripheral sensory nerve fibers and neurons in the spinal dorsal horn, leading to the spontaneous discharge activities of neurons and sensitization to stimulation, which may be the foundation of neuropathic pain. In the present study, it was found that high glucose may have an effect on the JAK2/STAT3-CAV-1-NR2B signal pathway, and the cell experiment indicated that it may affect p-CAV-1 and p-NR2B in PC12 cells through the upregulation of p-STAT3 on BV2 cells.

Several observations have confirmed that high glucose has a major role in the upregulation of p-JAK2 and p-STAT3 in mesangial cells [37]. Mao et al. [38] found that the high-glucose-induced JAK/STAT signaling pathway is activated in human glomerular mesangial cells. In addition, under high-glucose conditions, the phosphorylation of CAV-1 was significantly increased in podocytes [39]. Furthermore, CAV-1 expression has been observed in monolayer ECs exposed to high glucose [40]. Moreover, the expression of NR2B is also upregulated in retinal ganglion cells-5 under high-glucose conditions [41]. A positive correlation between poor control of blood glucose and severity of neuropathy or the risk and intensity of neuropathic pain was also detected [42]. Nevertheless, this correlation is not a linear relationship as some patients have severe neuropathy but do not develop neuropathic pain.

In the present in vivo experiments, 56% of T2DM rats successfully progressed to DNP, while the remaining T2DM rats did not present with neuropathic pain. Compared with the DNP group, the expression levels of p-JAK2, p-STAT3, CAV-1, and p-NR2B in the spinal dorsal horn in the no-DNP group were not significantly different, and this was the same with the Con group. Moreover, there was no difference in blood glucose levels between the DNP group and no-DNP group at all time points, and all remained at a high level. Therefore, in contradiction with the results of the in vitro experiments, the in vivo experiments confirmed that hyperglycemia did not affect the expression of p-JAK2, p-STAT3, CAV-1, and p-NR2B.

The following reasons may contribute to the differences observed in vivo and in vitro: (1) The expression of proteins was detected in certain cells in vitro, while this was detected in all cells in the spinal dorsal horn in vivo. (2) The concentration of glucose in the high-glucose group (33.3 mM) in vitro was pretty approximate to the blood glucose detected in T2DM rats (30.2 ± 4.8 mM). However, compared with the simple environment in vitro experiment, the results induced by high glucose in vitro may not be consistent with the results under the complex organism environment, involving multiple systems, organs and multiple cells in the in vivo experiment. It was also considered that the high blood glucose level under the complex environment in vivo may have some effects on the expression of some proteins in the JAK2/STAT3-CAV-1-NR2B signal pathway in certain cells of the spinal cord. However, this was not significant, and this pathway may be more correlated to the pathogenesis of neuropathic pain, rather than merely high blood glucose.

In vitro, the high glucose environment can significantly affect the expression of related proteins of the JAK2/STAT3-CAV-1-NR2B pathway. In vivo, high blood glucose levels have no significant effect on the expression of the protein, but each protein of the DNP rat spinal dorsal horn were abnormally expressed, and the JAK2 specific inhibitors could inhibit the above abnormal expressions in vivo and in vitro. Each protein of the JAK2/STAT3-CAV-1-NR2B pathway plays an important role in the pathogenesis of DNP. However, the specific signal transduction between the upstream and downstream of CAV-1 needs to be further confirmed.

## Abbreviations

T2DM: Type-2 diabetic mellitus
DNP: Painful diabetic neuropathy
NMDA: N-methyl-d-aspartate receptor
DRG: Dorsal root ganglion
JAK2: Janus Kinase 2
STAT3: Signal transducers and activators of transcription
CAV-1: Caveolin-1
NR2B: NMDA receptor 2B
HFSD: High-fat-sugar diet
STZ: Streptozocin
MWT: Mechanical withdrawal threshold
TWL: Thermal withdrawal latency
DMSO: Dimethylsulfoxide
AG-490: Tyrphostin AG490
CCI: Chronic constrictive injury
ISI: Insulin sensitivity index
BDNF: Brain-derived neurotrophic factor
